# Lynch syndrome–associated ultra-hypermutated pediatric glioblastoma mimicking a constitutional mismatch repair deficiency syndrome

**DOI:** 10.1101/mcs.a003863

**Published:** 2019-10

**Authors:** Chen Yang, Frances Austin, Hope Richard, Michael Idowu, Vernell Williamson, Fernanda Sabato, Andrea Ferreira-Gonzalez, Scott A. Turner

**Affiliations:** 1 Department of Pathology, Virginia Commonwealth University, Richmond, Virginia 23284, USA;; 2 Department of Pediatrics, Virginia Commonwealth University, Richmond, Virginia 23284, USA

**Keywords:** neoplasm of the central nervous system

## Abstract

Pediatric glioblastoma multiforme (GBM) has a poor prognosis as a result of recurrence after treatment of surgery and radiochemotherapy. A small subset of pediatric GBMs presenting with an ultra-high tumor mutational burden (TMB) may be sensitive to immune checkpoint inhibition. Here we report a 16-yr-old male with an ultra-hypermutated GBM. After incomplete surgical resection, molecular analysis of the tumor identified unusually high numbers of mutations and intratumor heterogeneity by a hotspot next-generation sequencing (NGS) panel. Further comprehensive molecular profiling identified a TMB of 343 mutations/Mb. An ultra-hypermutation genotype in pediatric GBMs is suggestive of a constitutive mismatch repair deficiency syndrome (CMMRD), which often acquires additional somatic driver mutations in replicating DNA polymerase genes. Tumor sequencing identified two *MSH6* nonsense variants, a hotspot *POLE* mutation and a mutational signature supportive of a germline MMR deficiency with a somatic *POLE* mutation. However, constitutional testing identified only one nonsense *MSH6* variant consistent with a Lynch syndrome diagnosis. This case represents the first confirmed Lynch syndrome case mimicking CMMRD by manifesting as an ultra-hypermutated pediatric GBM, following somatic mutations in *MSH6* and *POLE*. These findings permitted the patient's enrollment in an anti-PD-1 clinical trial for children with ultra-hypermutated GBM. Immunotherapy response has resulted in the patient's stable condition for over more than 1 year postdiagnosis.

## INTRODUCTION

Pediatric glioblastoma multiforme (GBM) is associated with a poor prognosis and is a common cause of death among children diagnosed with brain cancer (Durno et al. 2012; Ostrom et al. 2018). Approximately 7% of GBMs are diagnosed in children ages 0–19 yr with a median time of survival after recurrence of <6 mo (Durno et al. 2012). Although GBMs are rare in the pediatric population, the high rates of morbidity and mortality highlight the need for more effective treatments (Ostrom et al. 2018).

The use of immune checkpoint blockage has led to recent breakthroughs in treatment across various cancer types. These therapies include treatment with monoclonal antibodies targeting the programmed cell death-1 (PD-1) receptor or its ligand PD-L1. PD-L1 is an immune checkpoint receptor expressed on tumor cells that induces anergy by binding the PD-1 on activated T lymphocytes. Immune checkpoint inhibitors (e.g., anti-PD-1 antibody pembrolizumab) disrupt the immune evasion mechanisms adopted by tumor cells and are FDA-approved for a growing list of cancer types including melanoma and non-small-cell lung cancer (NCCN Guidelines). However, multiple clinical trials have shown that immune checkpoint inhibitors are not effective in GBM treatment (Filley et al. 2017). This may be due to the low tumor mutational burden (TMB) reported in the majority of GBMs (∼96.5%) (Alexandrov et al. 2013; Shlien et al. 2015; Bouffet et al. 2016; Hodges et al. 2017). The recognition of neoantigens derived from somatic nonsynonymous mutations by T lymphocytes is crucial for the effectiveness of immune checkpoint inhibition (McGranahan et al. 2016). Higher TMB and mismatch repair deficiencies lead to increased numbers of neoantigens and are now recognized as biomarkers for anti-PD-1/PD-L1 efficacy (Snyder et al. 2014; Le et al. 2015, 2017; Rizvi et al. 2015; Yarchoan et al. 2017; Hellmann et al. 2018).

A small group of pediatric GMBs contain the highest TMB reported in human cancer which result from germline biallelic mismatch repair defects and mutations in replicating DNA polymerase genes *POLE* or *POLD1* (Shlien et al. 2015; Bouffet et al. 2016; Campbell et al. 2017). Constitutional (or biallelic) mismatch repair deficiency syndrome (CMMRD) is a rare and often underdiagnosed cancer predisposition syndrome caused by the functional loss of both alleles in one of the MMR genes (*MLH1*, *MSH2*, *MSH6*, or *PMS2*) in an autosomal recessive manner (Bakry et al. 2014; Vasen et al. 2014; Wimmer et al. 2014; Durno et al. 2017). Nearly 100% of CMMRD patients develop cancers during their lifetime, most commonly GBM, hematologic malignancies, and gastrointestinal cancers, often in the first two decades of life (Hegde et al. 2005; Wimmer and Etzler 2008; Shlien et al. 2015; Campbell et al. 2017). A single heterozygous pathogenic germline variant in one of the MMR genes is diagnostic of Lynch syndrome (Boland et al. 2008). Lynch syndrome patients have an increased risk for colorectal/extracolorectal cancer (including GBMs) development typically in the third to sixth decades of life (Giardiello et al. 2014).

Pediatric GBMs with CMMRD exhibit ultra-high nonsynonymous mutations (>250/Mb) because of the complete ablation of two replication repair mechanisms, a biallelic germline MMR deficiency, and a somatically acquired proofreading defect in replicating DNA polymerase, most commonly in the *POLE* gene (Shlien et al. 2015; Campbell et al. 2017). The ultra-high TMB and the mutational signatures (predominantly NpCpG > NpTpG and NpCpT > NpApT) in these pediatric GBMs are unique and suggestive of CMMRD diagnosis with somatic *POLE* mutation (Shlien et al. 2015; Campbell et al. 2017). The prognosis for CMMRD cancers is generally poor, and the progression rate is typically faster than in Lynch syndrome–associated cancers (Campbell et al. 2017; Durno et al. 2017). However, cases of pediatric CMMRD-associated GBMs have shown a favorable response to immune checkpoint blockade (Bouffet et al. 2016; Larouche et al. 2018). Similarly, a case of a hypermutated GBM due to a germline POLE deficiency responded favorably to pembrolizumab (Johanns et al. 2016).

Here we report a 16-yr-old male who presented with GBM with intratumor mutational heterogeneity and an ultra-high TMB. The detection of two nonsense *MSH6* variants, a pathogenic *POLE* variant in tumor, and a mutational signature unique for MMR first\POLE second deficiency raised the concern for CMMRD. However, MMR immunohistochemical staining and germline testing confirmed a diagnosis of Lynch syndrome, making this the first reported case of a Lynch-associated tumor mimicking CMMRD. The ultra-hypermutation status of the GBM qualified this young patient for a clinical trial with the anti-PD-1 antibody pembrolizumab.

## RESULTS

### Case Presentation

A 16-yr-old Caucasian male was referred to Virginia Commonwealth University (VCU) Neurosurgery for 1 mo of persistent headaches. MRI showed a heterogeneous right parietal mass of 6.6 × 5.1 × 4.2 cm with significant surrounding edema, raising concern for a high-grade tumor of the cerebral cortex. Two days later, incomplete resection of the mass was performed. The immediate postsurgical MRI demonstrated residual enhancement measuring 1.9 × 0.8 × 0.9 cm. MRI at 5 wk postresection showed regrowth of residual neoplasm measuring 2.4 × 1.9 × 2.3 cm.

Per consensus review at adult and pediatric tumor boards, radiation therapy (59.4–60 Gy in 30–33 fractions) was administered in conjunction with concurrent temozolomide (90 mg/m2/dose daily) as first-line treatment for 42 d between 6–12 wk postresection. The patient tolerated the treatment well with no signs of hepatotoxicity, renal toxicity, or bone marrow suppression. Intermittent headache, myalgia, and radiochemotherapy-induced nausea and vomiting were treated. An MRI 4 wk postcompletion of radiochemotherapy, 16 wk postresection, showed enhancement that could not be differentiated from treatment-induced pseudoprogression.

The patient's family history was unremarkable. The patient's father is healthy and the mother has hypertension, history of seizures, achalasia, and noncancerous polyps. The patient's 21-yr-old sister is healthy with no identified medical issues. Extended family history showed no history of Lynch syndrome–associated tumors.

### Genomic Analyses

**Figure 1.**
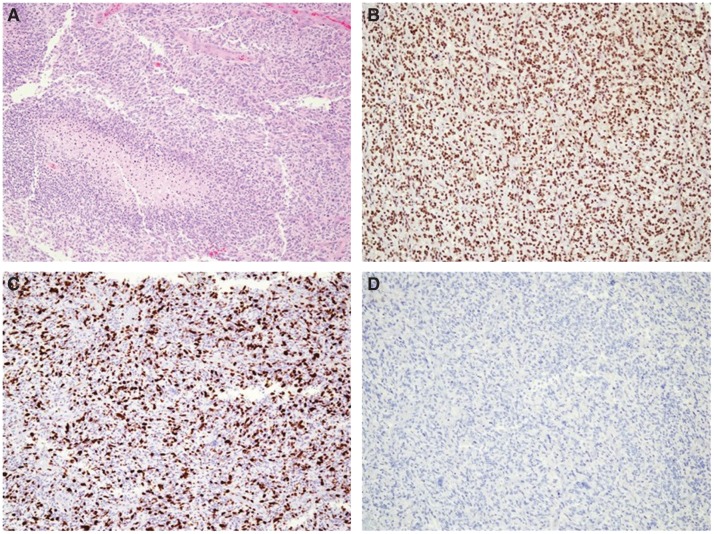
H&E and immunohistochemistry (IHC) staining in the case of pediatric GBM. (*A*) H&E stain showing endothelial proliferation and pseudopalisading necrosis, (*B*) ATRX IHC showing retained nuclear expression, (*C*) Ki67 IHC showing increased proliferation rate (60%), (*D*) IDH1 (R132H) IHC showing negative tumor staining. All photos were captured at 40× magnification.

The intraoperative surgical pathology microscopic interpretation was high-grade glioma. Hematoxylin and eosin (H&E) staining showed moderate nuclear atypia and pleomorphism. There were areas with oligo-like phenotype, including rounded nuclei with perinuclear halos and thin branching capillary vasculature. There were also scattered large atypical multinucleated cells, numerous mitotic figures, endothelial proliferation, and pseudopalisading necrosis (Fig. 1A). Further tumor characterization was performed (on block 2B): ATRX staining showed retained nuclear expression (Fig. 1B); Ki67 highlighted an overall increased (60%) proliferation rate (Fig. 1C); IDH1-R132H mutation stained negative by IHC (Fig. 1D), whereas GFAP and synaptophysin were positive by IHC; 19q and 1p deletion testing was negative by FISH; H3F3A-K27M mutation was negative by IHC; and MGMT gene promoter methylation was undetected by methylation-specific polymerase chain reaction (PCR) (data not shown). Following workup, the final pathologic diagnosis was WHO grade IV GBM.

**Figure 2.**
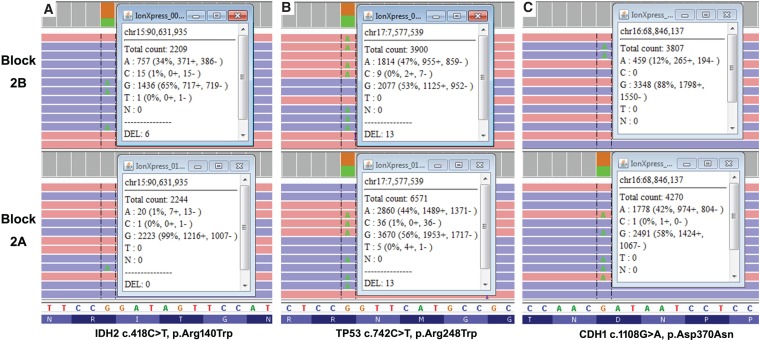
Comparison of variant allele frequency (VAF) for three nonsynonymous variants between Block 2B and 2A, in a case of pediatric GBM. The mean depth of coverage for blocks was >2000×. VAF < 3% (e.g., IDH2 c.418C>T, p.Arg140W) was manually calculated based on raw sequencing data in IGV.

A tissue specimen (block 2B) with 95% viable tumor was submitted to the VCU Molecular Diagnostic Laboratory for next-generation sequencing (NGS). The Oncogenomics Dx One Plus NGS assay, a tumor hotspot panel that can detect mutations from 50 oncogenes and tumor-suppressor genes using the Ion-Torrent S5XL platform, identified six pathogenic variants and eight variants of unknown significance (VUSs) for a total of 14 variants with variant allele frequency (VAF) of >3% (Table 1). Previous glioblastomas tested with this panel (*n* = 43) identified no more than four pathogenic variants or VUSs. Therefore, a second hotspot NGS was performed on a different block (block 2A, 95% viable tumor) from the same resection. Similarly, a total of 15 nonsynonymous variants were detected (Table 1). Interestingly, there were only six shared nonsynonymous variants, four of which had consistent VAF between 40%–50% (*SMAD4* p.Arg361His, *TP53* p.Ala161Thr, *TP53* p.Arg248Trp, *PDGFRA* p.Ser566Ile) in both blocks (Table 1). The eight variants called only in block 2B were confirmed to be either absent or at very low VAF (∼1%) in block 2A by manual review of BAM files on Integrative Genomics Viewer (IGV v2.4) (Fig. 2). Similarly, the nine variants called only in block 2A were confirmed to be absent in block 2B by manual sequence data review. Therefore, in addition to the high mutational load, substantial intratumor heterogeneity was observed in this case.

**Table 1.** Nonsynonymous mutations identified in a case of pediatric glioblastoma multiforme (GBM)

No.	Pathogenicity	Gene Name (transcripts)	cDNA coding	Protein coding	Block 2B	Block 2A
					VAF (%)	VAF (%)
1	Pathogenic	*IDH2* (NM_002168.3)	c.418C>T	p.Arg140Trp	34.8	0.9% not called
2	Pathogenic	*PIK3CA* (NM_006218.2)	c.323G>A	p.Arg108His	38.8	0.8% not called
3	Pathogenic	*PIK3CA* (NM_006218.2)	c.3127A>G	p.Met1043Val	38.2	1.0% not called
4	Pathogenic	*SMAD4* (NM_005359.5)	c.1082G>A	p.Arg361His	47.3	43.0
5	Pathogenic	*TP53* (NM_000546.5)	c.742C>T	p.Arg248Trp	46.9	43.8
6	Pathogenic	*TP53* (NM_000546.5)	c.481G>A	p.Ala161Thr	50.0	44.5
7	VUS	*SMARCB1* (NM_003073.4)	c.121G>T	p.Gly41Cys	5.8	0% not called
8	VUS	*KRAS* (NM_033360.3)	c.370A>G	p.Thr124Ala	6.8	0% not called
9	VUS	*CDH1* (NM_004360.3)	c.1108G>A	p.Asp370Asn	11.1	42.6
10	VUS	*HNF1A* (NM_000545.5)	c.806C>T	p.Ala269Val	11.2	40.9
11	VUS	*KDR* (NM_002253.2)	c.3629C>T	p.Pro1210Leu	11.2	1.1% not called
12	VUS	*KDR* (NM_002253.2)	c.1435C>T	p.Pro479Ser	20.6	0% not called
13	VUS	*HRAS* (NM_005343.2)	c.218G>A	p.Arg73His	32.2	0.8% not called
14	VUS	*PDGFRA* (NM_006206.4)	c.1697G>T	p.Ser566Ile	47.1	45.0
15	VUS	*ATM* (NM_000051.3)	c.3902A>G	p.Glu1301Gly	0% not called	29.1
16	VUS	*VHL* (NM_000551.3)	c.380G>A	p.Gly127Glu	0% not called	14.9
17	VUS	*HRAS* (NM_005343.2)	c.178G>T	p.Gly60Cys	0% not called	5.9
18	VUS	*CTNNB1* (NM_001904.3)	c.32A>G	p.Asp11Gly	0% not called	5.8
19	VUS	*CDH1* (NM_004360.3)	c.238G>A	p.Asp80Asn	0% not called	3.5
20	VUS	*PTPN11* (NM_002834.3)	c.165G>T	p.Lys55Asn	0% not called	3.4
21	VUS	*JAK3* (NM_000215.3)	c.2188G>A	p.Asp730Asn	0% not called	3.1
22	VUS	*APC* (NM_000038.5)	c.4376C>A	p.Thr1459Asn	0% not called	3.1
23	VUS	*APC* (NM_000038.5)	c.2663C>T	p.Ala888Val	0% not called	3.0

Next-generation sequencing was performed on a tissue block (Block 2B, with 95% viable tumor) using the Oncogenomic Dx One Plus Test. The high number of mutations detected was confirmed on a second block from the same resection (Block 2A, with 95% viable tumor). The Oncogenomic Dx One Plus Test can reliably call variants at a variant allele frequency (VAF) of 3% at 2000× coverage. Variants with a VAF <3% were manually called and VAFs were calculated based on raw data in Integrated Genome Viewer (IGV) for comparison purposes only.

(VUS) Variant of unknown significance.

The tumor on block 2A was further investigated by using the FoundationOne CDx comprehensive panel (targeting 324 genes), which identified 62 pathogenic variants and 326 VUSs in 189 genes with VAF > 1% (Table 2). Comparison analysis determined higher allele frequency (>40%) mutations were consistently observed across all tests. The comprehensive panel identified three additional clinically relevant variants with VAFs of 40%–50%: two *MSH6* nonsense mutations p.Gln4* and p.Tyr642* and a hotspot *POLE* pathogenic variant p.Ala456Pro located on the exonuclease domain of DNA polymerase ε. Among the genes with a mutation detected, NF1 has the highest number of pathogenic variants (six total), and the alterations (three nonsense mutations, one splicing donor change, and two common missense mutations) have low VAFs (3.5%–15.3%), consistent with the growth advantage from NF1 defects in the later stage of GBM development (Philpott et al. 2017).

**Table 2.** Results of hotspot versus comprehensive solid tumor testing for a case of pediatric GBM

	Hotspot panel	Comprehensive panel
Number of genes testing	50	324
Pathogenic mutations reported	6	64
Total mutations reported	15	326
Tumor mutational burden	N/A	343 muts/Mb
Pathogenic variants (>40% VAF) reported:	*SMAD4* p.A361H	*SMAD4* p.R361H
	*TP53* p.A161T	*TP53* p.A161T
	*TP53* p.R248W	*TP53* p.R248W
		*MSH6* p.Y642*
		*MSH6* p.Q4*
		*POLE* p.A456P

NGS was performed using the Oncogenomic Dx One Plus Test hotspot panel and the FoundationOne CDx comprehensive panel (Foundation Medicine). The total number of mutations, tumor mutational burden, and pathogenic variants of high frequency (>40% VAF) were compared.

Comprehensive testing also identified a tumor mutational burden (TMB) of 343 mutations/Mb and was classified as TMB-high (Table 2). Here, TMB counted only base substitutions (including synonymous variants) and indels of VAF > 5% in the coding region (∼1.1 Mb) of targeted genes after filtering out both the germline variants (known in population databases or predicted by the somatic-germline-zygosity algorithm) and cancer-driving alterations (Chalmers et al. 2017) (FoundationOne CDx FDA label). The threshold for TMB-high was 20 mutations/Mb. Interestingly, the comprehensive panel also generated a microsatellite stable status based on analysis of 95 microsatellite loci (instead of the five or seven MSI loci currently in common use). In addition, Foundation Medicine PD-L1 IHC analysis showed a tumor proportion score of 0% (negative for PD-L1 expression) by Dako 22C3 pharmDx testing (data not shown).

**Figure 3.**
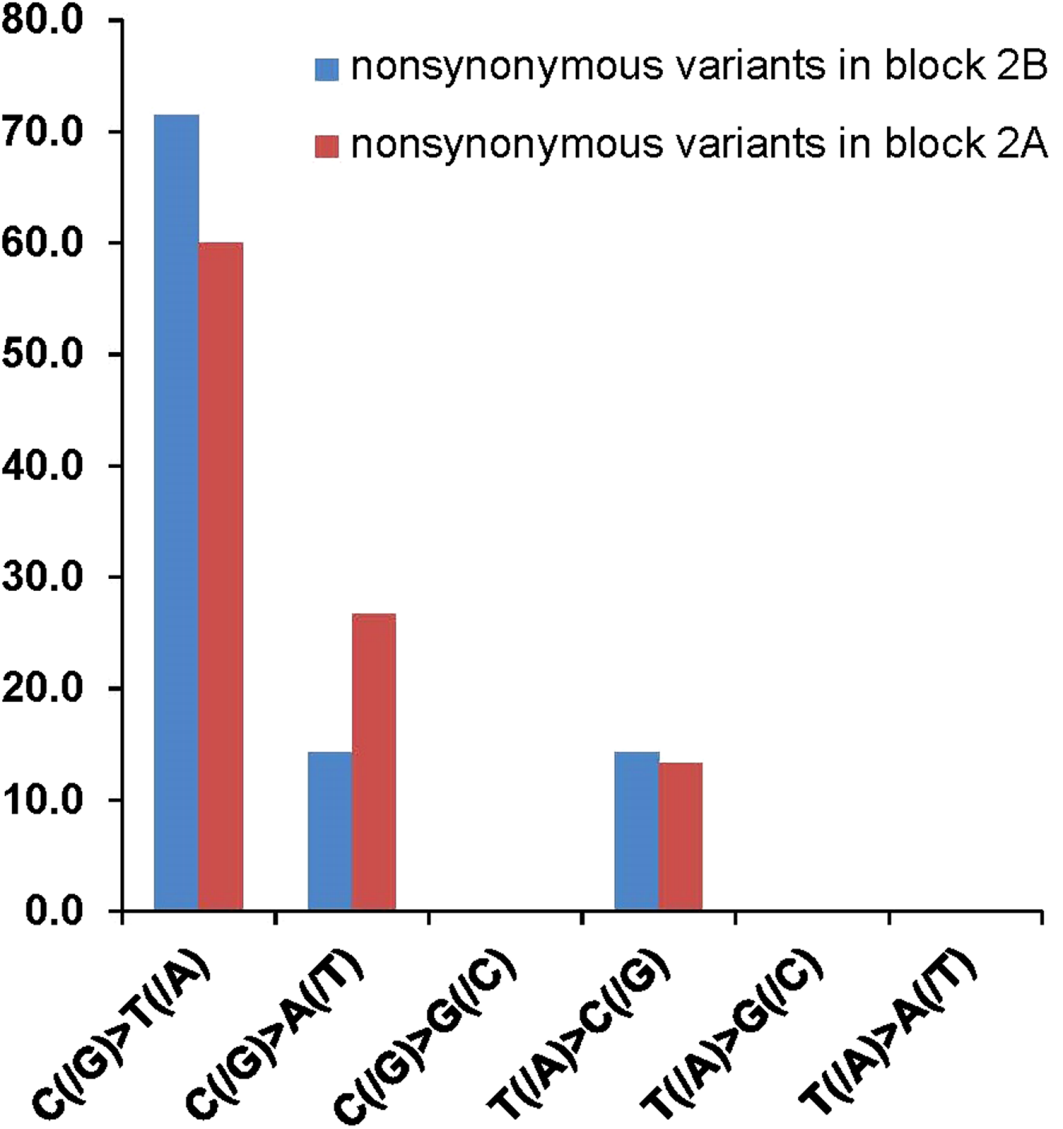
Mutational signature observed for a case of pediatric GBM. The nucleotide alteration pattern with predominant C>T (50% in the sequence context of NpCpG) and C>A (85.7% in the sequence context of NpCpT) and T>C fits well with the unique mutational signature specific for initiating MMR deficiency followed by acquired POLE mutation (MMR first/POLE second).

The ultra-high TMB in pediatric GBM has been statistically (*P* < 10^−13^) linked with the synergistic effect of germline MMR deficiency (i.e., CMMRD) and proofreading function defect from a somatic mutation in replicating DNA polymerase POLE or PLOD1 (Shlien et al. 2015; Hodges et al. 2017). Specific mutational signatures provide insight on the potential order of driving mutations in carcinogenesis (Alexandrov et al. 2013; Shlien et al. 2015; Hodges et al. 2017). Rereview of sequence variants in the hotspot panel illustrated a nucleotide alteration pattern with predominant C>T (50% in sequence context of NpCpG) and C>A (85.7% in sequence context of NpCpT) and T>C (Fig. 3), and an absence of the other possible alteration types. This mutational profile fits with the unique mutational signature specific for CMMRD followed by somatic *PLOE* mutation (MMR first/POLE second) (signature 14 in Alexandrov et al. 2013 and cluster 1 in Campbell et al. 2017). This mutational signature did not fit an MMR deficiency–only model (signature 6 or cluster 2) that largely lacks C>A mutations or a germline *POLE* mutation model (signature 10 or cluster 3) that has C>A mutations almost exclusively in the context of TpCpT. Given these findings, it was suspected that the patient had CMMRD, and the ultra-hypermutated GBM developed following a somatically acquired *POLE* exonuclease domain mutation.

**Figure 4.**
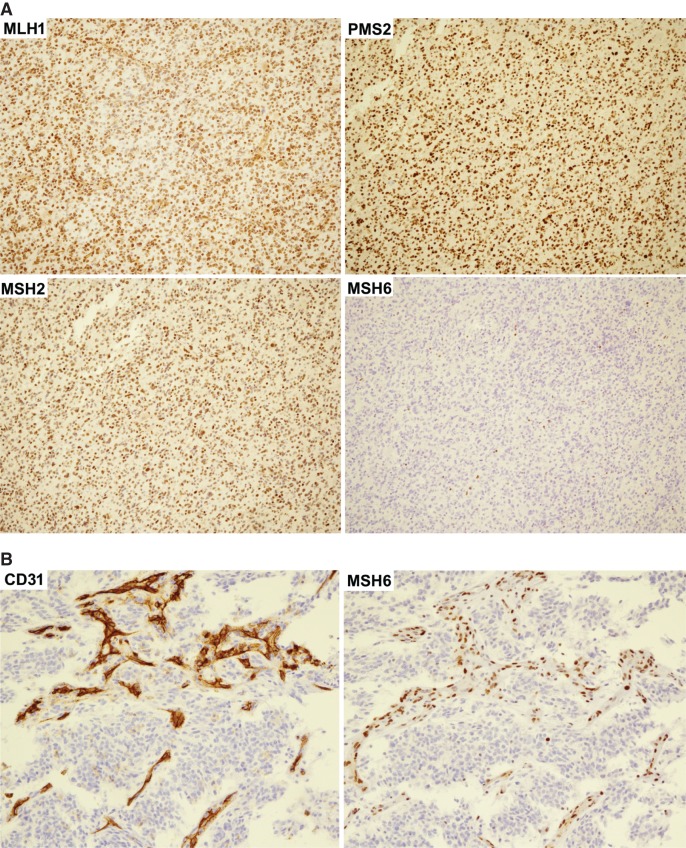
IHC staining for mismatch repair (MMR) proteins in a case of pediatric GBM. (*A*) Tumor nuclei stained positive for MLH1, PSM2, and MSH2, but negative for MSH6. There were scattered nontumor nuclei stained positive for MSH6. (*B*) To clarify the MSH6 protein expression in normal cells, adjacent FFPE slides were stained for the endothelial cell marker CD31 and for nuclear MMR protein MSH6. The nuclei of cells which stained positive for CD31 also stained positive for MSH6. All photos were captured at 40× magnification.

Following the recommendation of Europe and International CMMRD consortium (Bakry et al. 2014; Wimmer et al. 2014), MMR immunohistochemical staining was obtained to clarify the diagnosis. MLH1 (mAb# M1), MSH2 (mAb# G219-1129), and PMS2 (mAb# A16-4) proteins were detected in tumor cell nuclei, whereas MSH6 (mAb# SP93) stained negative in all tumor cell nuclei, suggesting the two nonsense *MSH6* variants are in the *trans* phase in the tumor cells. However, scattered nontumor nuclei showed positive nuclei staining for MSH6 (Fig. 4A). Adjacent FFPE slides with more vascularization were stained for CD31 and MSH6 to clarify the MSH6 expression in normal cells. The nuclei of CD31 positive endothelial cells showed MSH6 staining (Fig. 4B). MSH6 staining is suggestive of at least one expressing MSH6 allele and therefore this case is more consistent with a possible Lynch syndrome than CMMRD despite the observed TMB results.

Follow-up germline testing with an inherited cancer NGS panel on a peripheral blood specimen detected only the *MSH6* c.10C>T (p.Gln4*) variant. There was no evidence of other cancer predisposition variants initially identified in the tumor (*MSH6* p.Tyr642*, *POLE* p.Ala456Pro, *SMAD4* p.Arg361His, *TP53* p.Ala161Thr, or *TP53* p.Arg248Trp), confirming the unusual diagnosis of Lynch syndrome in this patient. Further targeted MSH6 testing in the family identified a germline *MSH6* p.Gln4* in the patient's father, whereas his mother and sister are negative for both *MSH6* variants. The father is now being routinely screened following Lynch syndrome guidelines (Giardiello et al. 2014).

Despite the diagnosis of Lynch syndrome, the ultra-hypermutation pattern of this patient's GBM qualified him for the clinical trial protocol PBTC-045C, pembrolizumab treatment for younger patients with hypermutated brain tumors (ClinicalTrials.gov Identifier: NCT02359565). Twelve courses (2 mg/kg i.v. at 3 wk interval) of pembrolizumab infusion have been performed at Children's National Medical Center Washington, District of Columbia. The trial will continue for up to 34 courses in the absence of significant toxicity or disease progression. At a year postdiagnosis, this patient remains in a clinically and radiologically stable condition. He has resumed normal activities including regular school attendance, marching band, and driving lessons.

## DISCUSSION

Pediatric CMMRD patients presenting with brain tumors commonly have biallelic loss of *PSM2* or *MSH6* (Bakry et al. 2014; Vasen et al. 2014; Wimmer et al. 2014), ultra-hypermutated pediatric GBM, and a unique MMR first/POLE second mutational signature (Shlien et al. 2015; Campbell et al. 2017). We presented a case with similar features but showing only one identified germline *MSH6* nonsense variant, diagnostic of Lynch syndrome and not the suspected CMMRD. Even though the suspected prevalence of germline susceptibility factors in pediatric and adult tumors has increased (Zhang et al. 2015; Mandelker et al. 2017), caution should continue to be applied when inferring germline status from tumor-only sequencing data (Shlien et al. 2015; Campbell et al. 2017).

This is the first report of a CMMRD-like Lynch syndrome case with a pediatric ultra-hypermutated GBM following a confirmed somatic second hit of a MMR and POLE mutation. We have since identified an additional reported case that mimicked CMMRD. In this case, a 13-yr-old developed an ultra-hypermutated malignant glioneuronal tumor and tumor sequencing identified two *PMS2* variants. A separate nonhypermutated glioma developed 2 yr later, and it was determined that the two tumors acquired distinct somatic second hits in the remaining *PMS2* allele (Wu et al. 2014). The mutational signature and potential somatic mutation in replication DNA polymerase genes in this case were not reported (Wu et al. 2014). This case helps illustrate the rare possibility that Lynch syndrome patients can develop malignant brain tumors at a young age. Confirmatory germline testing in these cases continues to be necessary to differentiate CMMRD form Lynch syndrome, because these two cancer predisposition syndromes have distinct prognosis and surveillance recommendations for the patients and substantially different inheritance risks to family members (Giardiello et al. 2014; Vasen et al. 2014; Tabori et al. 2017).

This case also illustrated that hotspot-targeted panels may identify cases of suspected ultra-high TMB. TMB measurements must be confirmed using a minimum of 1 Mb of sequencing to reliably detect levels of TMB typically observed across various tumor types. However, by initially using a hotspot panel in this case we were able to perform multiple targeted analysis on different tumor sections for a reduced cost generating potentially clinically useful information. First, the sequential hotspot sequencing illustrated both the high numbers of nonsynonymous variants and remarkable tumor heterogeneity, suggestive for high TMB. Second, through comparing sequencing results, pathogenic variants with consistently high VAFs across different blocks in one ultra-hypermutated tumor may be suggestive of potential driver mutations. Third, sequencing of multiple blocks in a tumor with high intratumor heterogeneity may minimize the risk of reporting misleading molecular information. As an example, one of the pathogenic variants observed through hotspot testing was *IDH2* p.Arg140Trp (35% in block 2B, <1% in block 2A, Fig. 2) and has a potential risk of being misinterpreted for its potential diagnostic and prognostic value. About 5% of GBM cases evolve from lower-grade astrocytoma with early *IDH* (i.e., *IDH1 and IDH2*) mutations, and these secondary GBMs usually have better prognosis than primary GBMs. *IDH1* p.Arg132His is the predominant *IDH* pathogenic variant in glioma (90%) and *IDH2* p.Arg140Trp is one of the hotspot variants commonly seen in acute myeloid leukemia and only rarely observed in gliomas (Andersson et al. 2011; Meggendorfer et al. 2018). If only block 2B was sequenced the high VAF of *IDH2* p.Arg140Trp may have been mistakenly interpreted as evidence for the secondary nature of this pediatric GBM with an *IDH* driver mutation. However, the fact that this mutation was not observed in other portions of the tumor calls into question the role of *IDH* as an early mutational event in this GBM.

Despite the unexpected diagnosis of Lynch syndrome as the underlying cancer susceptibility disorder, the mutational profile is still consistent with MMR first and POLE second in the tumor-initiating astrocyte. Interestingly, even though the *POLE* mutation was likely the result of MMR deficiency, the loss of POLE proofreading activity drives the late burst of mutagenesis and contributes more to the mutation pattern than the MMR deficiency itself (Shlien et al. 2015; Campbell et al. 2017). Therefore, although somatic MMR deficient cancers are usually microsatellite-unstable, ultra-hypermutated GBMs from CMMRD (or from Lynch syndrome in this case) with MMR first/POLE second are predominantly microsatellite-stable, similar to the ultra-hypermutated colorectal and endometrial cancers with somatic MMR and POLE proofreading deficiency (Cancer Genome Atlas 2012; Cancer Genome Atlas Research et al. 2013).

Ongoing clinical trials for immune checkpoint inhibition (ICI) have shown the potential for more effective management of properly selected GBM patients (Filley et al. 2017). Currently, it is not clear which ICI marker (TMB, PD-1/PD-L1 expression, or MMR deficiency) serves as a better indicator of response (Hodges et al. 2017). Multiple case reports have reported promising results in ultra-hypermutated GBM (Bouffet et al. 2016; Johanns et al. 2016; Larouche et al. 2018). In addition, a sustained response in a CMMRD patient after GBM recurrence was observed with combined checkpoint inhibition (Bouffet et al. 2016; Larouche et al. 2018). With survival typically >3 mo after GMB reoccurrence in pediatric CMMRD (Vasen et al. 2014; Westdorp et al. 2017), every effort to prevent or delay recurrence should be made.

The pediatric ultra-hypermutated GBM presented in this case had multiple poor prognostic markers: MMR first/ POLE second deficiency, *TP53* mutations, and lack of *MGMT* promoter methylation (Bouffet et al. 2016; Campbell et al. 2017). However, 8 mo after initiating immune-checkpoint inhibitor therapy, the patient had no signs of disease recurrence by MRI surveillance (monitored every 9 wk). Continued follow-up on this patient and reports of additional cases of ultra-hypermutated GBM will help determine the clinical utility of TMB measurement and the potential benefit of ICI treatment in these rare pediatric GBMs.

## METHODS

### Immunohistochemistry (IHC)

Immunohistochemistry was performed on FFPE block 2B cut with 3–5 µm thickness and stained according to the standard protocols by VCUHS surgical pathology laboratory. The monoclonal antibodies against human MLH1 (mAb# M1), MSH2 (mAb# G219-1129), PMS2 (mAb# A16-4), and MSH6 (mAb# SP93) were the VENTANA MMR IHC Panel from Roche, and the mouse antibody against CD31 (mAb# JC70) was from Sigma-Aldrich.

### 1p and 19q Deletion Testing FISH

The fluorescent in situ hybridization (FISH) studies assessing the deletions involving 1p36 and/or 19q13 were performed on FFPE block 2B with 5–7 µm thickness by VCUHS molecular cytogenetics laboratory, following the standard protocols. The 1p36 deletion status was determined by its signal ratio against the control probe 1q25, whereas the 19q13 deletion status was determined by its signal ratio against the control probe 19p13.

### Oncogenomics Dx One Plus NGS

Genomic DNA was extracted from FFPE tumor sections (block 2B and block 2A), each containing 95% neoplastic nuclei. The Ion AmpliSeq Cancer Hotspot Panel v.2 (Thermo Fisher) covers the hotspot regions in 50 frequently mutated oncogenes and tumor-suppressor genes. The raw signal data were analyzed using Torrent Suite v.4.? (Thermo Fisher) and the Torrent Suite v.5.1.0, Torrent Variant Caller v.5.2. Coverage metrics for the sequenced sample are provided in Table 3.

**Table 3.** Sequencing metrics of Ion Torrent Cancer Hotspot Panel

Coverage metrics	Block 2B	Block 2A
Total reads	837,191	760,498
Aligned reads	836,565	759,874
Reads on target	96.81%	98.79%
Mean read length	116 bp	116 bp
Average coverage (all targets)	3428×	3463×
Maximum coverage (all targets)	4863×	4545×
Minimum coverage (all targets)	251×	129×
Read coverage uniformity	98.06%	95.42%

### Reference Laboratory Testing

H3F3A-K27M mutation of tumor block 2B was determined by IHC (Cincinnati Children's Hospital), and MGMT gene promoter methylation status was measured of tumor block 2B by MGMT methylation-specific PCR assay (LabCorp). The comprehensive mutation profile of tumor block 2A was investigated with the FoundationOne CDx panel (FoundationOne), which detects point mutation, indel, copy-number alterations, and fusion detection in 324 cancer-related genes. The comprehensive testing also has the capability of calculating the TMB and the microsatellite status. TMB counted only base substitutions (including synonymous variants) and indels of VAF > 5% in the coding region (∼1.1 Mb) of targeted genes after filtering out both the germline variants (known in population databases or predicted by the somatic-germline-zygosity algorithm) and cancer-driving alterations (Chalmers et al. 2017). The microsatellite stable status was based on the analysis of 95 microsatellite loci. Foundation Medicine PD-L1 IHC analysis by Dako 22C3 pharmDx testing was used for determining PD-L1 expression level. Germline testing on a peripheral blood specimen was performed on the Common Hereditary Cancers Panel (test code 01102) at Invitae. Additional test information for the hereditary cancer panel is available at https://www.invitae.com/.

## ADDITIONAL INFORMATION

### Data Deposition and Access

A table of sequencing results from the Oncogenomics Dx One Plus NGS assay has been included. Per laboratory policy, the submission of variants to ClinVar requires additional patient consent, which is not typically obtained in tumor cases for clinical patient management. The submission of additional somatic and germline variants identified through reference laboratory testing was subject to individual reference laboratory policy. The germline *MSH6* p.Gln4* reported in this case has been reviewed by multiple ClinVar submitters (VCV000183723.4).

### Ethics Statement

All testing performed was part of clinical patient management and not performed on a research basis. As per the Virginia Commonwealth University Office of Research and Innovation and the Virginia Commonwealth University Institutional Review Board “medical case studies involving no more than two patients are not considered systematic investigation” and therefore do not require IRB approval.

### Author Contributions

C.Y. and S.A.T. conceived and drafted the manuscript. F.A. provided clinical patient management and contributed sections to the manuscript. H.R. and M.I. interpreted immunohistochemistry data, provided a pathological assessment of the case, and contributed to sections of the manuscript. V.W. and F.S. provided sequencing interpretation and contributed to technical sections of the manuscript. A.F.-G. provided clinical, pathological, and technical assessment of data and contributed to all sections of the manuscript.

### Funding

No external funding sources were used.

### Competing Interest Statement

The authors have declared no competing interest.

### Referees

S. Zheng

Jianling Ji

Anonymous
